# Supplementary material for: Phenotypic signatures of immune selection in HIV-1 reservoir cells
Source: Nature. 2023 Jan 4;614(7947):309–17. doi: 10.1038/s41586-022-05538-8 (PMC9908552; doi:10.1038/s41586-022-05538-8)
Supplement: Supplementary file 1 — Reporting Summary [file 41586_2022_5538_MOESM1_ESM.pdf]

nature portfolio

Corresponding author(s): Mathias Lichterfeld

Last updated by author(s): October 5th, 2022

Reporting Summary

Nature Portfolio wishes to improve the reproducibility of the work that we publish. This form provides structure for consistency and transparency in reporting. For further information on Nature Portfolio policies, see our Editorial Policies and the Editorial Policy Checklist.

Statistics

For all statistical analyses, confirm that the following items are present in the figure legend, table legend, main text, or Methods section.

n/a	Confirmed	
☐	☒	The exact sample size (*n*) for each experimental group/condition, given as a discrete number and unit of measurement
☐	☒	A statement on whether measurements were taken from distinct samples or whether the same sample was measured repeatedly
☐	☒	The statistical test(s) used AND whether they are one- or two-sided<br>*Only common tests should be described solely by name; describe more complex techniques in the Methods section.*
☒	☐	A description of all covariates tested
☐	☒	A description of any assumptions or corrections, such as tests of normality and adjustment for multiple comparisons
☐	☒	A full description of the statistical parameters including central tendency (e.g. means) or other basic estimates (e.g. regression coefficient) AND variation (e.g. standard deviation) or associated estimates of uncertainty (e.g. confidence intervals)
☐	☒	For null hypothesis testing, the test statistic (e.g. *F*, *t*, *r*) with confidence intervals, effect sizes, degrees of freedom and *P* value noted<br>*Give P values as exact values whenever suitable.*
☒	☐	For Bayesian analysis, information on the choice of priors and Markov chain Monte Carlo settings
☒	☐	For hierarchical and complex designs, identification of the appropriate level for tests and full reporting of outcomes
☒	☐	Estimates of effect sizes (e.g. Cohen's *d*, Pearson's *r*), indicating how they were calculated

Our web collection on statistics for biologists contains articles on many of the points above.

Software and code

Policy information about availability of computer code

Data collection	Tapestri (MissionBio, v2.0.1)
Data analysis	Bioconductor (https://bioconductor.org/, version 3.14),<br>Biorender (https://biorender.com),<br>Cutadapt (https://cutadapt.readthedocs.io/en/stable/, version 2.5),<br>Graphpad prism (https://www.graphpad.com/scientific-software/prism/, version 8.2.1),<br>Highlighter (https://www.hiv.lanl.gov/content/sequence/HIGHLIGHT/highlighter_top.html),<br>MAFFT (https://mafft.cbrc.jp/alignment/software, version 7),<br>MEGA (https://www.megasoftware.net, version 7.0.26),<br>MUSCLE (http://www.drive5.com/muscle, version 3.8.1551),<br>Python (https://www.python.org, version 3.6),<br>R (https://www.r-project.org, version 4.1),<br>SPICE (https://niaid.github.io/spice/, version 6.0),<br>bwa (https://github.com/lh3/bwa, version 0.7.12),<br>monocle3 (https://cole-trapnell-lab.github.io/monocle3/, version 1.0.0),<br>samtools and bcftools (http://www.htslib.org/, version 1.9),<br>Adobe Illustrator (https://www.adobe.com/products/illustrator.html, version 2022 (26.1)),<br>Flow Jo (V10.7),<br>DiVa (V08.01),<br>Leiden Community detection algorithm from Monocle v3 (https://cole-trapnell-lab.github.io/monocle3/, version 1.0.0)

For manuscripts utilizing custom algorithms or software that are central to the research but not yet described in published literature, software must be made available to editors and reviewers. We strongly encourage code deposition in a community repository (e.g. GitHub). See the Nature Portfolio guidelines for submitting code & software for further information.

Data

Policy information about availability of data

All manuscripts must include a data availability statement. This statement should provide the following information, where applicable:
- Accession codes, unique identifiers, or web links for publicly available datasets
- A description of any restrictions on data availability
- For clinical datasets or third party data, please ensure that the statement adheres to our policy

Owing to study participant confidentiality concerns, viral sequencing data cannot be publicly released, but will be made available to investigators upon reasonable request and after signing a data sharing agreement. Correspondence and requests for materials should be addressed to Dr. Mathias Lichterfeld (mlichterfeld@partners.org).

Field-specific reporting

Please select the one below that is the best fit for your research. If you are not sure, read the appropriate sections before making your selection.

☒ Life sciences ☐ Behavioural & social sciences ☐ Ecological, evolutionary & environmental sciences

For a reference copy of the document with all sections, see nature.com/documents/nr-reporting-summary-flat.pdf

Life sciences study design

All studies must disclose on these points even when the disclosure is negative.

Sample size	A total of n=4 ART-treated participants, and n=1 EC were analyzed in data described in Figure 1-4 (peripheral blood viral reservoir cells). Data from n=3 study participants are described in Figure 5 (lymph node reservoir cells). No computational approach was used to determine these sample sizes, testing was based on availability of more than 1 million memory CD4 T cells per study participant.
Data exclusions	No data from the described individuals were excluded.
Replication	Multiple microfluidic cartridges were run for each patient sample. In selected cases (n=14), the proviral library from a given cartridge was sequenced twice, with similar results.
Randomization	No randomization was performed, because we performed an analysis of study participants enrolled in an observational study.
Blinding	Coded samples from study participants were used throughout the study; laboratory personnel was not blinded with regard to the respective study subjects, since this was a non-interventional, observational study.

Reporting for specific materials, systems and methods

We require information from authors about some types of materials, experimental systems and methods used in many studies. Here, indicate whether each material, system or method listed is relevant to your study. If you are not sure if a list item applies to your research, read the appropriate section before selecting a response.

Materials & experimental systems

n/a	Involved in the study	
☐	☒	Antibodies
☒	☐	Eukaryotic cell lines
☒	☐	Palaeontology and archaeology
☒	☐	Animals and other organisms
☐	☒	Human research participants
☒	☐	Clinical data
☒	☐	Dual use research of concern

Methods

n/a	Involved in the study	
☒	☐	ChIP-seq
☐	☒	Flow cytometry
☒	☐	MRI-based neuroimaging

Antibodies

Antibodies used	All antibodies were purchased from Biolegend, BD and eBioscience. All antibodies and clone names are provided in the methods section of the manuscript. A cocktail of lyophilized antibodies from Biolegend was used for proteogenomic profiling experiments which contained the following antibodies: PD-L1 (clone 29E.2A3), CD276 (clone DCN.70), HVEM (clone122), CD155 (clone SKII.4), CD154/CD40L (clone 24-31), CCR4 (clone L291H4), PD-1 (A17188B), TIGIT (clone A15153G), CD44 (clone BJ18), CXCR3 (clone G025H7), CCR5 (clone HEK/1/85a), CCR6 (clone G034E3), CXCR5 (clone J252D4), CCR7 (clone G043H7), KLRB1/CD161 (clone HP-3G10), CTLA-4 (clone BNI3), LAG-3 (clone 11C3C65), KLRG1 (clone 14C2A07), CD95 (clone, DX2), OX40/CD134 (clone Ber-ACT35), CD57 (clone HNK-1), TIM-3 (clone F38-2E2), BTLA/CD272 (clone MIH26), CD244/2B4 (clone 2-69), IL-2R (clone TU27), CD137/4-1BB

(clone 4B4-1), GITR/CD357 (clone 108-17), CD28 (clone CD28.2), CD127 (clone A019D5), IL-6R (clone UV4), HLA-E (clone 3D12), MICA/B (clone 6D4), IL-15R/CD215 (clone JM7A4), IL-21R (clone 2G1-K12), TNFR2 (clone 3G7A02), CD160 (clone BY55), LIGHT/CD258 (clone T5-39), IL-10R/CD210 (clone 3F9), TGFB-R (clone W17055E), IL-12R (clone S16020B), CD6 (clone BL-CD6), CD49d (clone 9F10), CD25 (clone BC96), CD30 (clone BY88), CD69 (clone FN50), CD45RA (clone HI100), CD38 (clone HIT2), HLA-DR (clone L243), CD4 (clone RPA-T4), CD2 (clone TS1/8), CD3 (clone UCHT1), CD62L (clone DREG-56), CD45RO (clone UCHL1). IgG2a (Biolegend, clone MOPC-173, catalog 400299), IgG2b (Biolegend, clone MPC-11, catalog 400383).

The following antibodies were used for cell sorting: CD3-PerCP-Cy5.5 (BioLegend, clone UCHT1, catalog 300430), CD3-FITC (BioLegend, clone UCHT1, catalog 300406), CD4-BUV395 (BD, clone RPA-T4, catalog 564724), CD4-BV711 (BioLegend, clone RPA-T4, catalog 300558), PD1-FITC (BioLegend, clone A17188B, catalog 621612), TIGIT-BV421 (BioLegend, clone A15153G, catalog 372710), PVR-PE (eBioscience, clone 2H7CD155, catalog 12-1550-41), HVEM-APC (Biolegend, clone 122, catalog 318808), BTLA-FITC (Biolegend, clone MIH26, catalog 344524), KLRG1-APC (Biolegend, clone 14C2A07, catalog 368606), HLAE-BV421 (Biolegend, clone 3D12, catalog 342612), PDL1-PE (Biolegend, clone MIH2, catalog 393608)

Validation

All antibodies were validated by manufacturers. Validation information is available for each antibody at www.biolegend.com, https://www.bd.com/en-us, and https://www.thermofisher.com/us/en/home/life-science/antibodies/ebioscience.html.

The lyophilized antibody cocktail from Biolegend was validated by Biolegend.

CD3-PerCP-Cy5.5 (BioLegend, clone UCHT1, catalog 300430)
Reactivity: Human
Host Species: Mouse
Application: FC - Quality tested
Application Notes: Additional reported applications (for the relevant formats) include: immunohistochemical staining of acetone-fixed frozen sections4,6,7 and formalin-fixed paraffin-embedded sections11, immunoprecipitation1, activation of T cells2,3,5, Western blotting9, and spatial biology (IBEX)16,17. The LEAF™ purified antibody (Endotoxin < 0.1 EU/µg, Azide-Free, 0.2 µm filtered) is recommended for functional assays (Cat. No. 300413, 300414, and 300432). For highly sensitive assays, we recommend Ultra-LEAF™ purified antibody (Cat. No. 300437, 300438, 300465, 300466, 300473, 300474) with a lower endotoxin limit than standard LEAF™ purified antibodies (Endotoxin < 0.01 EU/µg).
Application References: Salmeron A, et al. 1991. J. Immunol. 147:3047. (IP)
Graves J, et al. 1991. J. Immunol. 146:2102. (Activ)
Lafont V, et al. 2000. J. Biol. Chem. 275:19282. (Activ)
Ryschich E, et al. 2003. Tissue Antigens 62:48. (IHC)
Thompson AG, et al. 2004. J. Immunol. 173:1671. (Activ)
Sakkas LI, et al. 1998. Clin. Diagn. Lab. Immun. 5:430. (IHC)
Mack CL, et al. 2004. Pediatr. Res. 56:79. (IHC)
Thakral D, et al. 2008. J. Immunol. 180:7431. (FC) PubMed
Van Dongen JJM, et al. 1988. Blood 71:603. (WB)
Yoshino N, et al. 2000. Exp. Anim. (Tokyo) 49:97. (FC)
Pollard, K. et al. 1987. J. Histochem. Cytochem. 35:1329. (IHC)
Luckashenak N, et al. 2013. J. Immunol. 190:27. PubMed

CD3-FITC (BioLegend, clone UCHT1, catalog 300406)
Reactivity: Human
Host Species: Mouse
Application: FC - Quality tested
Application Notes: Additional reported applications (for the relevant formats) include: immunohistochemical staining of acetone-fixed frozen sections4,6,7 and formalin-fixed paraffin-embedded sections11, immunoprecipitation1, activation of T cells2,3,5, Western blotting9, and spatial biology (IBEX)16,17. The LEAF™ purified antibody (Endotoxin < 0.1 EU/µg, Azide-Free, 0.2 µm filtered) is recommended for functional assays (Cat. No. 300413, 300414, and 300432). For highly sensitive assays, we recommend Ultra-LEAF™ purified antibody (Cat. No. 300437, 300438, 300465, 300466, 300473, 300474) with a lower endotoxin limit than standard LEAF™ purified antibodies (Endotoxin < 0.01 EU/µg).
Application References: Salmeron A, et al. 1991. J. Immunol. 147:3047. (IP)
Graves J, et al. 1991. J. Immunol. 146:2102. (Activ)
Lafont V, et al. 2000. J. Biol. Chem. 275:19282. (Activ)
Ryschich E, et al. 2003. Tissue Antigens 62:48. (IHC)
Thompson AG, et al. 2004. J. Immunol. 173:1671. (Activ)
Sakkas LI, et al. 1998. Clin. Diagn. Lab. Immun. 5:430. (IHC)
Mack CL, et al. 2004. Pediatr. Res. 56:79. (IHC)
Thakral D, et al. 2008. J. Immunol. 180:7431. (FC) PubMed
Van Dongen JJM, et al. 1988. Blood 71:603. (WB)
Yoshino N, et al. 2000. Exp. Anim. (Tokyo) 49:97. (FC)
Pollard, K. et al. 1987. J. Histochem. Cytochem. 35:1329. (IHC)
Luckashenak N, et al. 2013. J. Immunol. 190:27. PubMed

CD4-BUV395 (BD, clone RPA-T4, catalog 564724)
Reactivity: Human (QC Testing)
Host Species: Mouse
Application: Flow cytometry (Routinely Tested)

CD4-BV711 (BioLegend, clone RPA-T4, catalog 300558)
Reactivity: Human
Host Species: Mouse
Application: FC - Quality tested
Application Notes: The RPA-T4 antibody binds to the D1 domain of CD4 (CDR1 and CDR3 epitopes) and can block HIV gp120 binding and inhibit syncytia formation. Additional reported applications (for the relevant formats) include: immunohistochemistry of

acetone-fixed frozen sections3,4,5, blocking of T cell activation1,2, and spatial biology (IBEX)10,11. This clone was tested in-house and does not work on formalin fixed paraffin-embedded (FFPE) tissue. The Ultra-LEAF™ purified antibody (Endotoxin < 0.01 EU/µg, Azide-Free, 0.2 µm filtered) is recommended for functional assays (Cat. No. 300569 - 300574).
Application References: Knapp W, et al. 1989. Leucocyte Typing IV. Oxford University Press. New York.
Moir S, et al. 1999. J. Virol. 73:7972. (Activ)
Deng MC, et al. 1995. Circulation 91:1647. (IHC)
Friedman T, et al. 1999. J. Immunol. 162:5256. (IHC)
Mack CL, et al. 2004. Pediatr. Res. 56:79. (IHC)
Lan RY, et al. 2006. Hepatology 43:729.
Zenaro E, et al. 2009. J. Leukoc. Biol. 86:1393. (FC) PubMed
Yoshino N, et al. 2000. Exp. Anim. (Tokyo) 49:97. (FC)
Stoeckius M, et al. 2017. Nat. Methods. 14:865. (PG)
Radtke AJ, et al. 2020. Proc Natl Acad Sci USA. 117:33455-33465. (SB) PubMed
Radtke AJ, et al. 2022. Nat Protoc. 17:378-401. (SB) PubMed

PD1-FITC (BioLegend, clone A17188B, catalog 621612)
Reactivity: Human
Host Species: Mouse
Application: FC - Quality tested
Application Notes: A17188B antibody can block the binding of NAT105 and EH12.2H7 antibodies to the target.

TIGIT-BV421 (BioLegend, clone A15153G, catalog 372710)
Reactivity: Human
Host Species: Mouse
Application: FC - Quality tested
Application Notes: This clone can suppress anti-CD3 induced T cell proliferation in vitro based on in-house testing. This clone has been tested in-house and determined to not be suitable for applications in immunohistochemistry of paraffin-embedded tissue sections (IHC-P).
Additional reported applications (for the relevant formats) include: Blocking
Application References: Stamm H, et al. 2018. Oncogene. Pubmed

PVR-PE (eBioscience, clone 2H7CD155, catalog 12-1550-41)
Reactivity: Human
Host Species: Mouse
Application: Flow Cytometry (Flow)

HVEM-APC (Biolegend, clone 122, catalog 318808)
Reactivity: Human
Host Species: Mouse
Application: FC - Quality tested
Application Notes: The 122 antibody has been shown to be useful for flow cytometry, Western blot, and ELISA.
Application References: Cheung TC, et al. 2010. J. Immunol. 185:1949. PubMed
Hobo W, et al. 2012. J Immunol. 189:39. PubMed.

BTLA-FITC (Biolegend, clone MIH26, catalog 344524)
Reactivity: Human
Host Species: Mouse
Application: FC - Quality tested
Application Notes: Additional reported applications (for the relevant formats) include: inhibition of T cell proliferation and cytokine production1. Clone MIH26 has agonistic activity on BTLA, resulting in the inhibition of activation.
Application References: Otsuki N, et al. 2006. Biochem. Bioph. Res. Co. 344:1121.
Okano M, et al. 2008. Clin. Exp. Allergy 38:1891.

KLRG1-APC (Biolegend, clone 14C2A07, catalog 368606)
Reactivity: Human
Host Species: Mouse
Application: FC - Quality tested

HLAE-BV421 (Biolegend, clone 3D12, catalog 342612)
Reactivity: Human
Host Species: Mouse
Application: FC - Quality tested
Application References: Lee N, et al. 1998. Proc. Natl. Acad. Sci. USA. 95:5199.
Wooden SL, et al. 2005. J. Immunol. 175:1383.
Monaco EL, et al. 2008. J. Immunol. 181:5442.
Corrah TW, et al. 2011. J. Virol. 85:3367.

PDL1-PE (Biolegend, clone MIH2, catalog 393608)
Reactivity: Human
Host Species: Mouse
Application: FC - Quality tested

Human research participants

Policy information about studies involving human research participants

Population characteristics	Clinical characteristics of the study participants are shown in the Extended Data Figure 1A-B and the Extended Data Figure 9A-B
Recruitment	All study persons were recruited based on referral by HIV clinicians and infectious disease physicians. The enrollment protocols allowed recruited of men and women >18 years old, of any race or ethnicity. Patients were included in our prior studies and selected for this project according to the following criteria: availability of sufficient cells for experiments, availability of full-genome sequencing data and proviral integration site data, and relatively high frequency of genome-intact HIV-1 proviruses in CD4 T cells.
Ethics oversight	The MassGeneralBrigham Human Research Committee approved all sample collection at MGH and BWH; the IRB of the NIH supervised sample collection at the NIH Clinical Center.

Note that full information on the approval of the study protocol must also be provided in the manuscript.

Flow Cytometry

Plots

Confirm that:

☒ The axis labels state the marker and fluorochrome used (e.g. CD4-FITC).

☒ The axis scales are clearly visible. Include numbers along axes only for bottom left plot of group (a 'group' is an analysis of identical markers).

☒ All plots are contour plots with outliers or pseudocolor plots.

☒ A numerical value for number of cells or percentage (with statistics) is provided.

Methodology

Sample preparation	As described in the methods section of the manuscript.
Instrument	FACS Aria cell sorting device
Software	DiVa version V08.0l<br>FlowJo Version Vl0.7
Cell population abundance	Purity of sorted cell populations was >90%.
Gating strategy	The lymphocyte population was identified based on FSC/SSC characteristics, followed by identification of singlets on an FSC-Area vs FSC-Height plot. Viable cells were identified using live/dead viabiliy dye. The remaining gating strategy is shown in Extended Data Figure 6 and 7.

☒ Tick this box to confirm that a figure exemplifying the gating strategy is provided in the Supplementary Information.
